# Decision level scheme for fusing multiomics and histology slide images using deep neural network for tumor prognosis prediction

**DOI:** 10.1038/s41598-025-09869-0

**Published:** 2025-07-15

**Authors:** Tingting Zhao, Yongyong Ren, Hui Lu, Yan Kong

**Affiliations:** 1 Center for Biomedical Informatics, Shanghai Engineering Research Center for Big Data in Pediatric Precision Medicine, Shanghai Children’s Hospital, Shanghai, 200240 China; 2 SJTU-Yale Joint Center for Biostatistics and Data Science, Institute of Translational Medicine, Shanghai Jiao Tong University, Shanghai, China; 3 Institute of Bioinformatics, Shanghai Academy of Experimental Medicine, Shanghai, China; 4 State Key Laboratory of Microbial Metabolism, Department of Bioinformatics and Biostatistics, School of Life Sciences and Biotechnology, Shanghai Jiao Tong University, Shanghai, China

**Keywords:** Multimodal data fusion, Pathological slide images, Deep learning, Multiomics, Prognosis, Cancer, Computational biology and bioinformatics

## Abstract

Molecular biostatistical workflows in oncology often rely on predictive models that use multimodal data. Advances in deep learning and artificial intelligence technologies have enabled the multimodal fusion of large volumes of multimodal data. Here, we presented a decision level multimodal data fusion framework for integrating multiomics and pathological tissue slide images for prognosis prediction. Our approach established the spatial map of instances by connecting the neighboring nuclei in space and calculated the characteristic tensor via graph convolution layers for the input pathological tissue slide images. Global Average Pooling was applied to align and normalize the feature tensors from pathological images and the multiomics data, enabling seamless integration. We tested our proposed approach using Breast Invasive Carcinoma data and Non-Small Cell Lung Cancer data from the Cancer Genome Atlas, which contains paired whole-slide images, transcriptome data, genotype, epienetic, and survival information. In a 10-fold cross-validation, the comparison results demonstrated that the multimodal fusion paradigm improves outcome predictions from single modal data alone with the average C-index increasing from 0.61 to 0.52 to 0.75 and 0.67 for breast cancer and non-small cell lung cancer cohort, respectively. The proposed decision level multimodal data fusion framework is expected to provide insights and technical methodologies for the follow-up studies.

**Supplementary Information:**

The online version contains supplementary material available at 10.1038/s41598-025-09869-0.

## Introduction

Cancer is caused by aberrant cell proliferation in human body and is defined by hallmark histopathology, genomic, and genotypic heterogeneity in the tumor micro-environment that contribute to variability in treatment response and survival rate^1,2^. Advances in sequence technologies and bio-informatics have sped up the widespread of multiomics data in cancer research for biomarkers, prognosis, and therapeutic response prediction^3,4^. Morover, the digital pathology and deep convolution neural networks have promoted the potential applications to analyze gigapixel whole slide images for lesion detection, benign and malignant discrimination, and prognosis prediction^5,6^. The integration of multimodal data presents more opportunities on precision oncology and provides possibilities to increase the robustness and precision of diagnostic and predictive models^7^. Recent developments in bioinformatics platforms facilitate the comprehensive analysis of multiomics data, bridging the gap between genomic, transcriptomic, and imaging modalities to provide deeper insights into tumor heterogeneity and treatment response^8^. Such advancements bring AI closer to mimicking human intelligence in clinical decision-making^9^.

In the field of prognostic prediction, data-driven approaches have shown remarkable power in informing clinical decisions^10^. Combining whole-slide pathological images with genomic data has been shown to enhance the accuracy of prognostic predictions, as this integration can provide complementary information^11–13^. However, the current research on the integration of pathological images and genomic data remains insufficient, particularly in the in-depth exploration of multiomics data, with most studies limited to one or two types of omics data (e.g., mRNA)^13^.

In this paper, we proposed a decision level multimodal data fusion framework^14,15^ for fusing imaging features from whole slide images and molecular features from multiomics data which consist of expression data, methylation data, and the mutation data. To verify the improvement of model genuinely results from the fusion features, deep-learning-based prognostic prediction models were built for the single-modal data, ensuring consistency with the neural network architecture embedded in the multimodal fusion framework prior to the fusion module. Compared with the models based on either histology information or omic information, the proposed multimodal fusion network achieved the best performance when tested on both The Cancer Genome Atlas (TCGA)^16^ Breast Invasive Carcinoma (BRCA) data^17^ and Non-Small Cell Lung Cancer (NSCLC) data^18^. Fusing the spatial morphological information from pathological whole slides with the multiomics provides the possibility to better understand the associations between phenotype and genotype and to further explore the capabilities of image-omic assays for tumor diagnosis, treatment, prognosis, and drug resistance prediction.

## Results

### Network implementation details

All the models were implemented using the Python PyTorch (version 1.4.0) deep learning framework and trained using two Tesla V100 GPUs on a single node. Due to GPU memory constraints, we used stochastic gradient descent with a batch size of 20. The initial learning rate was set to 0.01 and then decreased by a factor of 10 every 10 epochs. The weights were initialized from a Gaussian distribution with a mean and standard deviation of 0 and 0.01, respectively. Models for single modal data were trained from scratch for a total of 50 epochs since there is no prior knowledge. Moreover, rather than training the multimodal fusion network end-to-end, we initialized the model using weights from the single-modal network. All models were trained and tested on the same configurations and run for about 10–16 h.

### Typical features from multimodal data

**Fig. 1:**
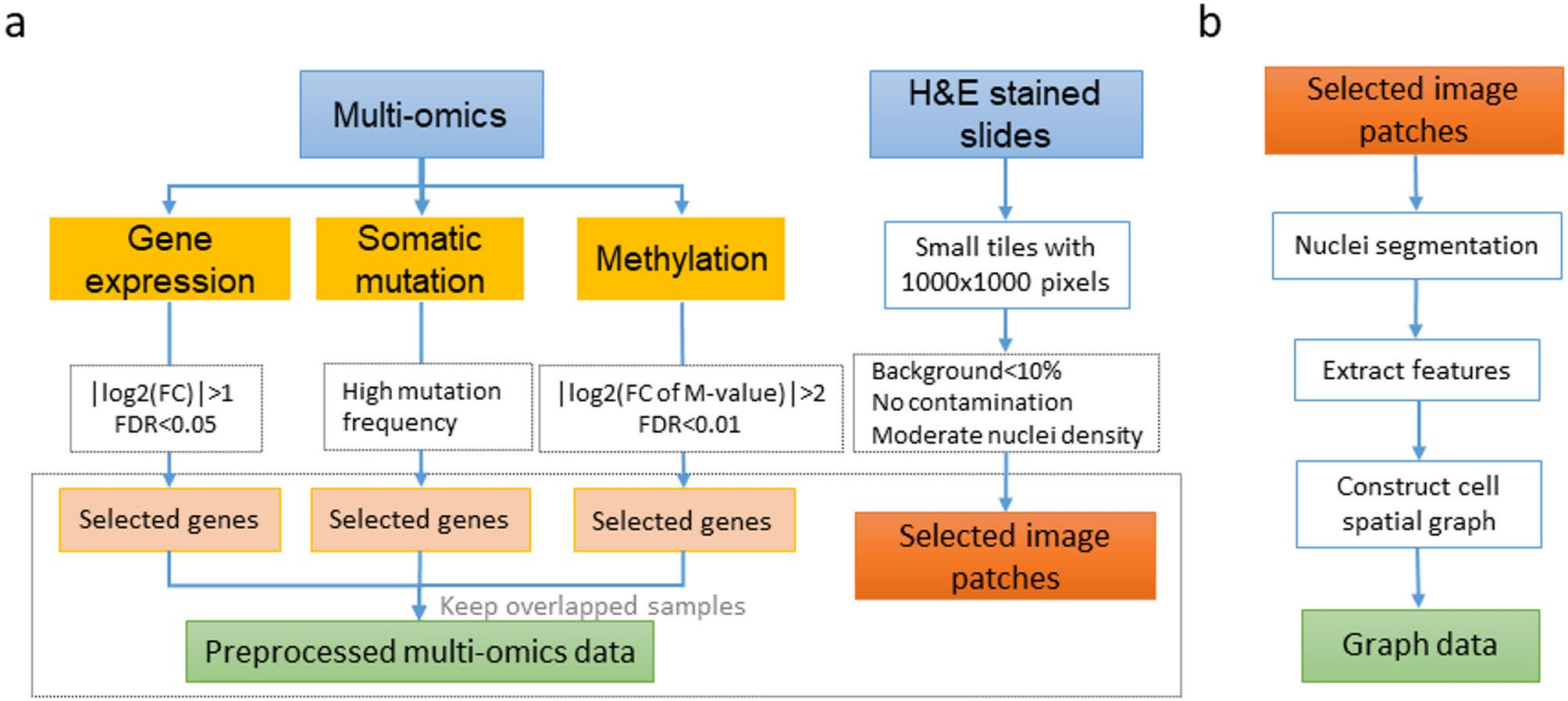
Details of feature extraction steps for different modal data. (**a**) Pre-process workflow of different modal data. (**b**) The steps of generating nuclei spatial graph data.

For the gene expression data, 1340 genes were selected using a cut-off of |log_2(_Fold Change)|>1 and false discovery rate (FDR) < 0.05. For the somatic mutation data, 4518 sites with high mutation frequency (appearing in over 80% of samples) were chosen. For the methylation data, 9305 sites that were hypermethylated or hypomethylated were selected using a cut-off of |log_2_(Fold Change of M-value)| >2 and FDR < 0.01. After intersecting the samples across data modalities, the multiomics data matrix was updated with 679 patients and the updated dimensions were as follows: 679 × 1,340 for expression data, 679 × 4,518 for mutation data, and 679 × 9,305 for methylation data. As shown in Fig. 1A, we got a multiomics feature-based tensor for each sample.

**Fig. 2:**
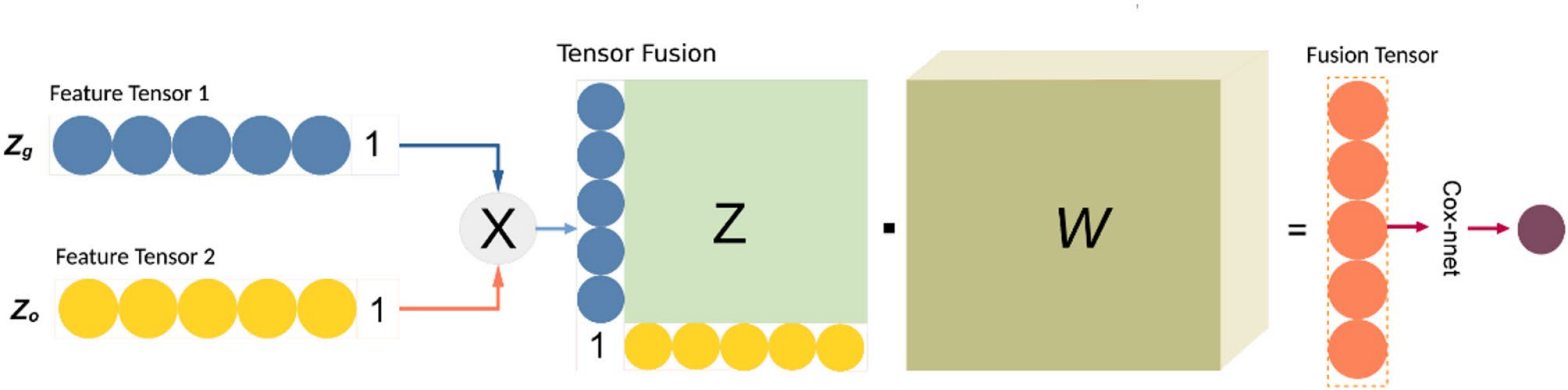
Tensor fusion method used in this study.

We used the hematoxylin and eosin (H&E) stained imaging as the second modality of data. Considering the large dimensions of the whole slide images, we selected 50 slide tiles of 1000 × 1000 pixels for each patient, resulting in a total of 33,950 image patches, along with the corresponding cellular spatial graph data. As shown in Fig. 1a and b, we finally got an image features-based tensor for each sample.

The fusion tensor was calculated using the tensor fusion method and was considered as the input for Cox-nnet for prognosis analysis (Fig. 2). The data mentioned here all pertain to the BRCA cohort, and the details of the samples and features used in this study were listed in Table 1.

**Table 1:** The data summary before and after pre-processing.

Cancer	Data type	Raw tumor samples	Raw normal samples	Filter condition	Selected samples	Selected features
BRCA	RNA-seq	1071	99	|log_2_FC|>2 and FDR < 0.05	679	1340
	Methylation site	774	81	|log_2(_FC of M-value)|>2 and pvalue < 0.05	679	9305
	Somatic mutation	989	–	Appeared in over 80% samples	679	4518
	Graphs of tissue slide	–	–	–	679 × 50	1041 (17 + 1024)
LUAD	RNA-seq	510	58	|log_2_FC|>2 and FDR < 0.05	443	2500
	Methylation site	451	32	|log_2(_FC of M-value)|>2 and pvalue < 0.05	443	8743
	Somatic mutation	567	–	appeared in over 80% samples	443	1577
	Graphs of tissue slide	–	–	–	443 × 50	1041
LUSC	RNA-seq	496	49	|log_2_FC|>2 and FDR < 0.05	359	2500
	Methylation site	365	41	|log_2(_FC of M-value)|>2 and pvalue < 0.05	359	8743
	Somatic mutation	485	–	appeared in over 80% samples	359	1577
	Graphs of tissue slide	–	–	–	359 × 50	1041

### Model performance comparison

**Fig. 3:**
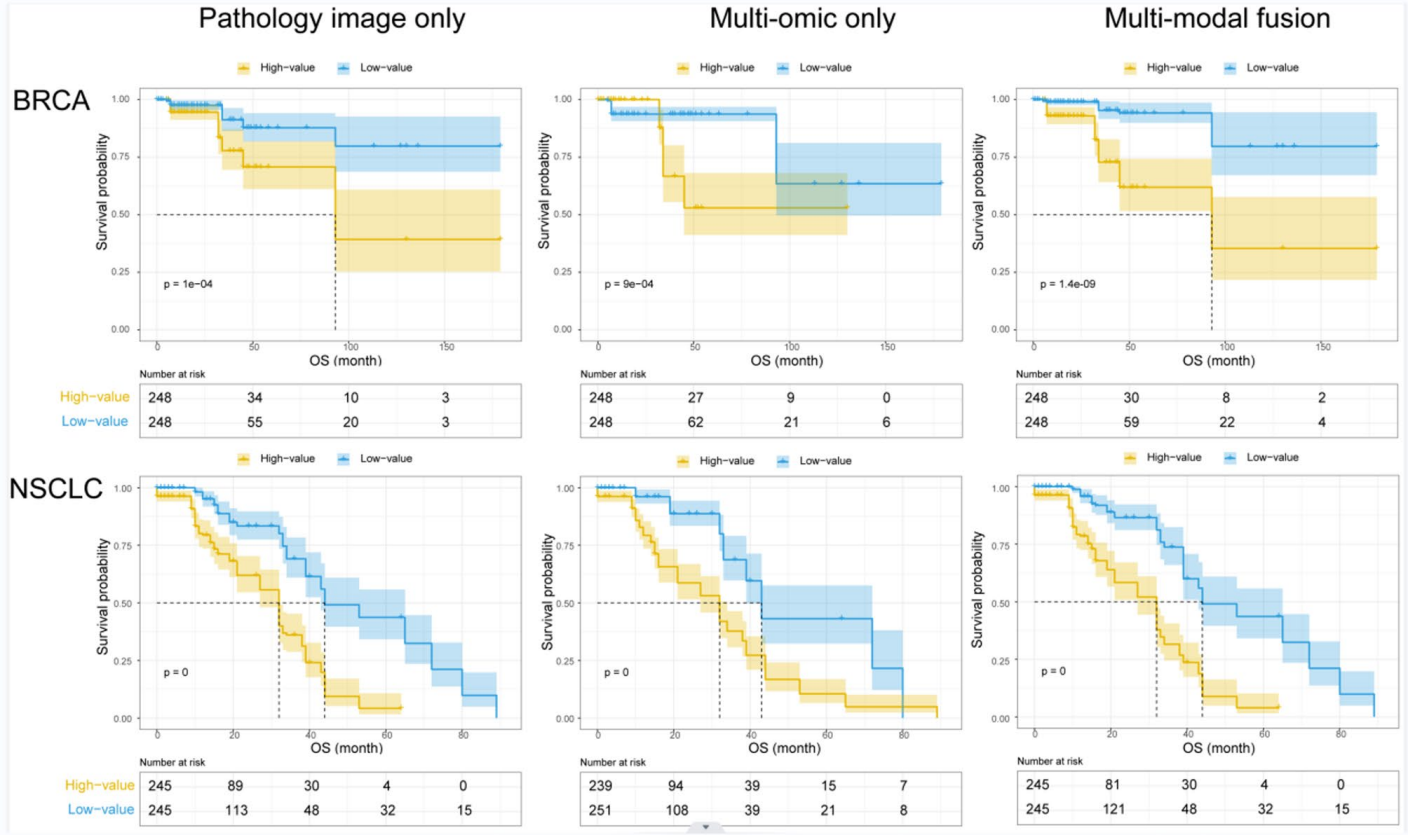
Kaplan-Meier curves for different risk groups predicted by 3 models.

Different prognostic prediction models were developed using only multiomics data, only slide image data, and the fusion data. In order to avoid overfitting, the model was trained and tested using 10 fold cross-validation. The predicted hazard ratios on 10 independent test datasets were added and averaged. We also built a baseline model using the cox regression function based on clinical characteristics, including clinical grade and tumor stage. To assess the prognostic accuracy of the prognostic prediction model using multimodal data, we performed the assessments using Kaplan-Meier method both on the BRAC and NSCLC datasets. Patients were stratified into high- or low-risk group according to the predicted survival time using the median value as the cutoff. The results demonstrated that the two groups were more distinct with the model that used the fusion data (Fig. 3).

Concordance index (C-index) for the prognostic prediction models were calculated and all the deep learning-based models outperformed the baseline model (see Tables 2 and 3). The average C-index for prognosis prediction of the multimodal fusion data model was higher than the single-modal data model for both NSCLC and BRCA dataset. Moreover, area under the curve (AUC) of the multimodal fusion model in the prediction of five-year disease-specific outcome was significantly higher than the multiomics-only model (Venkatraman’s p-value was 0.00113 for BRCA and 0.01267 for NSCLC) and pathology-only model (Venkatraman’s p-value was 0.00251 for BRCA and 0.00096 for NSCLC). Taken together, our proposed multimodal data fusion framework was significantly better in discriminating low-risk from high-risk patients when applied to both the BRCA (log-rank p-value < 0.05) and NSCLC (log-rank p-value < 0.01) datesets. We further demonstrate the extensibility and generalization performance of our fusion method through testing on 10 independent cross-validation test sets.

**Table 2:** Comparison of prognosis between 4 models for BRCA.

Model Name	C-index
Baseline COX (clinical)	0.586 ± 0.043
Graph Convolution Net (only graph)	0.610 ± 0.129
Multiomics Neural Net (only omics)	0.696 ± 0.153
Fusion Net	**0.753** ± 0.067

**Table 3:** Comparison of prognosis between 4 models for NSCLC.

Model name	C-index
Baseline COX (clinical)	0.553 ± 0.107
Graph Convolution Net (only graph)	0.515 ± 0.0143
Multiomics Neural Net (only multiple omics)	0.647 ± 0.0840
Fusion Net	**0.669** ± 0.0673

In multivariable analyses, the Fusion Net score remained a robust independent predictor of survival after adjusting for clinical covariates. In the BRCA cohort, the Fusion Net score demonstrated a strong independent association with survival outcomes in multivariable Cox regression (HR = 4.24, 95% CI: 3.04–5.91, *p* < 0.001), adjusted for age and tumor stage (Supplementary Table). While advanced tumor stages (e.g., Stage IV, HR = 6,27) showed expected high mortality risks, the Fusion Net score provided additional prognostic discrimination beyond traditional staging. In the NSCLC cohort, the Fusion Net score demonstrated a strong independent association with survival outcomes in multivariable Cox regression (HR = 1.75, 95% CI: 1.57–1.95, *p* < 0.001), adjusted for age, tumor stage and gender (Supplementary Table).

## Discussion

Computational oncology is experiencing unprecedented challenges and opportunities due to the accumulated multimodal data and the emerging deep learning based method^19,20^. A major goal of computational oncology is to predict the future states of tumors such as prognosis of patients^21^lymphatic metastasis^22^and the recurrent status^23^. More generally, the aim is to link data characteristics with tumor state prediction or clinical outcomes. Although multimodal data are widely used in various oncology research areas^24,25^few studies have applied them together to address clinical prediction problems especially using both multiomics and image data.

Images and omics data are two different modal data that represent the genotype and phenotype levels, respectively. Combiningimaging and omics data is not a trivial task, as these data are typically designed and generated for different purposes. Modality-specific characteristics and complementary features provide additional information and hold great potential for enhancing the power of statistical models. Therefore, fusing omics and images approaches is essential for combining the macro-level structural and functional data embedded in the biomedical images, with micro-level molecular signatures in omics, thereby providing a coherent, accurate, and reliable source of information. Integration of Omics and biomedical images is an emerging interdisciplinary field aimed at development of methods for integration of biomedical images and omics data. Some studies have suggested several prognostic risk factors, including deferentially expressed genes, somatic mutation sites^26^abnormal methylation modification sites^27^and the morphological features of tumor cells^28^. In our study, we proposed a decision level multimodal fusion framework for integrating multiomics data with pathological tissue slide images. The multiomics data we used included RNA-Seq expression, somatic mutations, and methylation profiles which covered genomic, transcriptomic and epigenetic information. With prognosis prediction as the goal, we compared the preformances of different models. Our proposed multimodal fusion network achieved better performance on both independent datasets. The results demonstrated that the modal complementary could improve the prediction performance. Furthermore, multimodal data fusion methodology could also be helpful in the tumor mechanism studies, such as genotype-phenotype relationships, invasive analyses, and the identification of new biomedical markers. The fusion strategy holds promise for improving current preventive and predictive disease models, enabling earlier detection, more precise diagnosis, and tailored treatment. Therefore, multimodal data fusion will be fundamental for the development of a personalized medicine approach, extremely beneficial for the patients as well as for the whole healthcare system. Our proposed decision level multimodal fusion framework is hoped to provide insights and technically methodologies for the follow-up studies that seed to merge heterogeneous data in disease mechanisms.

Despite these advantages, it is always rare to obtain all modal data at the same time. According to our comparison results, although multimodal fusion could improve the prediction power, the performance of the single-modal model is also acceptable (Fig. 3; Tables 2 and 3). In our study, we trained the multimodal fusion network on BRCA and NSCLC datasets separately. Following this framework, one can conduct training steps on any dataset to obtain a pretrained weight. To solve the problem of missing modalities while leveraging multimodal fusion, we suggest using the pretrained feature tensor for the missing modality.

For future works, it is attractive to incorporate the structured text data as another modal data, especially the records of medical care and related information, into our framework. In terms of the technical approach, the temporal point process (TPP) could be an effective tool for incorporating certain priors, such as self-exciting patterns, intothe record sequences^29,30^. In particular, the deep point process has emerged as a promising topicby combining the interpretability of classic Bayesian TPP and high capacity of neural models. It offers a pathway to integrate with our deep learning based multimodal learning and analysis framework.

**Fig. 4:**
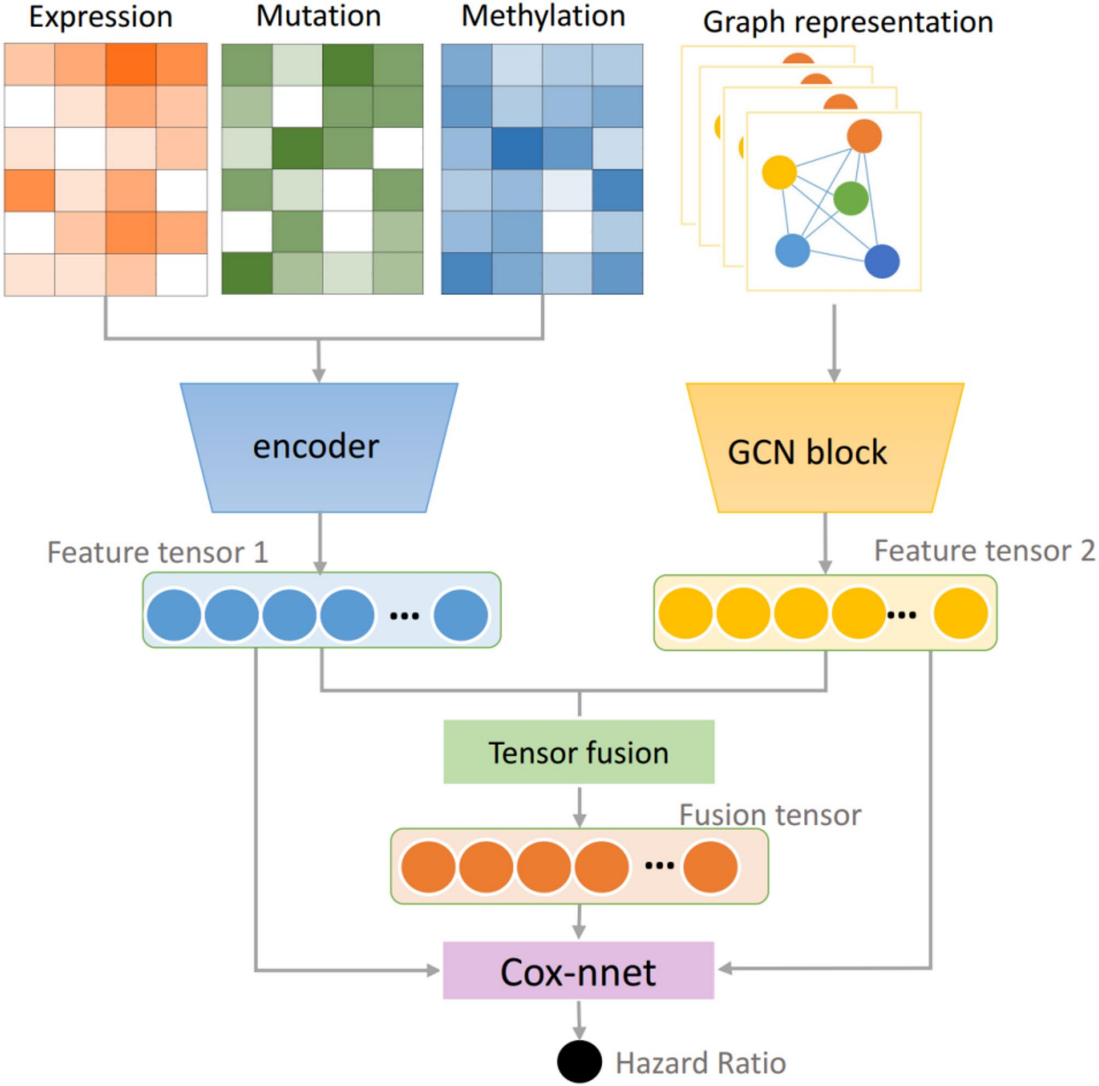
Overview of our proposed Decision level fusion strategy for multimodal data. The details of the GCN block is explained in Fig. 5.

## Methods

### Data description

The multimodal dataset used in this study are sourced from TCGA. We downloaded the multiomics data and H&E stained pathological tissue slide images of BRCA and NSCLC patients from the website. The multiomics data we used included RNA-Seq expression, somatic mutations, and methylation profiles. To construct the typical feature matrix within the matched samples, we use different pre-process pipelines for the multiomics data and the pathological tissue slide images (Fig. 1a). A total of 679 BRCA patients and 802 NSCLC patients were selected in this study, with the NSCLC dataset comprising two parts: 443 cases of Lung Adenocarcinoma (LUAD) and 359 cases of Lung Squamous Cell Carcinoma (LUSC). The correspondingly clinical pathological characteristics were also used, including age, sex, smoking history, stage, pathological subtype, the last follow-up days, and overall survival (OS) days.

For the RNA-Seq expression profiles, we extracted the mRNA expression matrix and calculate the different expression levels of genes between the tumor and adjacent normal tissues using the edgeR package^31^. The expression matrix of significantly differentially expressed genes of tumor tissues were retained as the first omic data. For the mutation data, we downloaded the publicly available masked somatic mutation results generated by mutect2^32^ software and arranged them into the mutation frequency matrix according to gene names. And for the methylation data, the beta-value matrix was downloaded as the source data, which was then transformed into an M-value matrix as suggested by Du et al.^33^. We performed the T-test and retained those significant methylated tags and their methylation profiles on tumor tissues. Patients who have all three types of omics data were conseved.

Due to the large size of high-resolution tissue slide images and the limitations of GPU memory, these images cannot be used directly. We randomly cropped 50 tiles with 1000 × 1000 pixel size for each patient using the OpenSlide package of python^34^. During the process of selection, we set some filtering criteria including background area less than 10%, without obvious contamination, and with moderate nuclei density. Small tiles were preserved if they met the criteria.

### The proposed fusion network

In this work, we proposed a decision level multimodal data fusion framework for multiomics and pathological tissue slide images for survival outcome prediction. Our proposed method models feature interactions across modalities by using the tensor fusion strategy and assess the expressiveness of each representation before fusion. The workflow of the decision level fusion framework is depicted in Fig. 4.

The tensor representations of pathological slide images and multiomics data are processed in parallel. Prior to the Tensor fusion module, the feature tensor of slide images was obtained via Graph Convolutional Network (GCN), and the feature tensor of the multiomics data was calculated using multi-layer perception. The Tensor fusion module will generate a fused tensor conducted as the input of the Cox-nnet.

### Prognosis prediction based on H&E slide images

The spatial information of cells in the tumor microenvironment, as observed in histopathology slide images, is critical for subtyping and prognosis prediction. Unlike feature representations from whole slide images using Convolutional Neural Networks, graph representations explicitly capture only specific preassigned features of each nucleus, which can be scaled to cover large regions of tissue. To construct graphs containing the micro-environment information, we first performed nuclei instance segmentation in the histopathology region of interest to define the graph nodes. Next, the adjacent nuclei were connected using the K-Nearest Neighbors to represent the edges among the graph nodes. In addition, the attributions for each nucleus we defined were captured. Finally, GCN was used for learning the robust representation of the entire graph for prognosis prediction.

#### Nuclei segmentation and feature extraction

We tried to focus on utilizing the spatial information and image features of the nuclei. The nuclei instances were segmented firstly using TSN-Unet^6^. And 17 morphological characteristics were extracted for each nucleus using the Python OpenCV package, including area, minor axis length, major axis length, radio of major inner circle, average values of RGB channels, average gray value, range of gray value, value range of RGB channels, the standard deviation of values from RGB channels, the standard deviation of gray value, the distance from the nearest cell. Besides the measurable morphological features, we also used the contrastive predictive coding^35^ to extract another 1024 features from 64 × 64 pixel size region centered around each nucleus. Next, we built the cell graph with the edge set generated by connecting the adjacent 5 neighbor cells using the K-Nearest Neighbors algorithm from the FLANN library. Finally, the graph structured data were constructed with each node represent a nuclei and the previous defined features were treated as the attributes of the node (Fig. 1b).

#### GCN for spatial graphs of nuclei in tissue slide images

In this study, we introduced a three-layer GCN. The GraphSAGE architecture^36^ was used in the aggregate and combine functions. The hierarchical structure of nuclei graphs was encoded by using the self-attention pooling strategy (SAGPooling)^36^which is a hierarchical pooling method that performs local pooling operations of node embeddings in a graph.

**Fig. 5:**
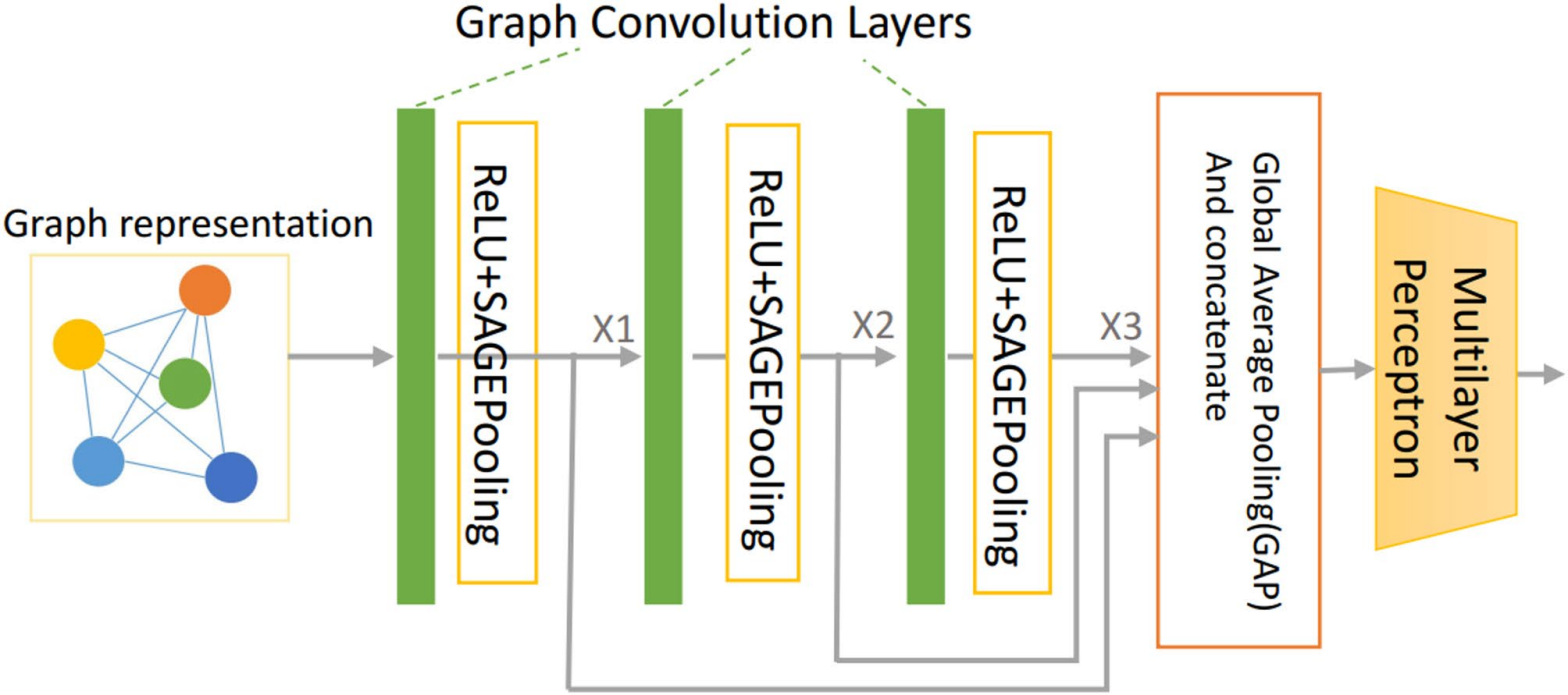
The detailed architecture of the proposed GCN for the spatial graphs of nuclei. The input is the original spatial graph of nuclei. The aggregate and combine functions are adopted from the GraphSAGE architecture. The self-attention pooling strategy, SAGPooling is adopted to encode the hierarchical structure of nuclei graphs.

As shown in Fig. 5, the input was the original spatial graph of nuclei. Three graph convolution layers were used in our proposed method, each employing ReLU as the activation function, followed by a single SAGPooling layer. During the training steps, the feature maps from one batch will be transformed into a vector representation. All three vectors generated from the 3 graph convolution layers correspondingly were concatenated. Then, a Global Average Pooling layer followed by a fully connected module was sequentially added to the network.

#### Prognosis prediction model based on nuclei Spatial graphs

The cox-nnet module was employed to calculate the Hazard Ratio for prognosis measurement. We got the feature tensor for slide imaging from the output of the fully connect layers, which we named *Z*_*g*_. The cox-nnet module^37^ uses the partial log-likelihood function [44] to calculate the loss, which is defined as:

$$\:\text{c}\text{o}\text{s}\text{t}=\sum\:({{\uptheta\:}}_{\text{i}}-\text{l}\text{o}\text{g}\sum\:{\text{e}}^{{{\uptheta\:}}_{\text{i}}}\text{*}\text{R})\text{*}\text{C}$$

where R indicates the adjacent function and is defined as:

$$\:{\text{R}}_{\text{i}\text{j}}=\left\{\begin{array}{c}1,{\text{t}}_{\text{i}}\le\:{\text{t}}_{\text{j}}\\\:0,{\text{t}}_{\text{i}}>{\text{t}}_{\text{j}}\end{array}\right.$$

While θ is the predicted hazard ratio of the patient, and C represents the survival status.

### Prognosis prediction based on multiomics data

Due to the difference in dimension and representation forms, the multiomics data used in this study were initially processed separately. At this stage, we employed a multi-layer perceptron to extract their feature representations. Then, the feature maps generated from three parallel multi-layer perceptrons were integrated via vector computation since they have the same dimension. The integrated tensor was also the feature tensor of the multiomics data, which we named *Z*_*o*_. Finally, the multiomics integrated tensor was sent into the cox-nnet to predict the hazard ratio.

### Prognosis prediction based on multimodal fusion data

We introduced the tensor fusion method^38^ in this study to intergratge two different modalities: multiomics data and the cell spatial graph derived from the tissue slide image. According to the separate process of the multiomics data and the tissue slide image data, we obtained the feature tensors for each modal- *Z*_*g*_ (dimension was 128) and *Z*_*o*_ (dimension was 32). The first step for the tensor fusion algorithm was to construct the input tensor Z (Z∈R^d1,d2^), which was generated by the cross product of *Z*_*g*_ and *Z*_*o*_ after adding 1 for each other. Then a vector representation (H_fusion_) was produced through a fully connected layer (Fig. 2).

$${{\text{H}}_{{\text{fusion}}}}={\text{g}}\left( {{\text{z}};{\text{W}},{\text{b}}} \right)\,=\,{\text{W}}\cdot{\text{Z}}\,+\,{\text{b}}$$

where h, b∈R^dg^, W denotes the weight matrix, and b is the deviant vector. Since Z is the two-order tensor, W is a tensor of order 3. The dimension of W is d_1_ × d_2_ × d_h_, where d_h_ represents the size of the output layer and d_h_ equals 1 in the prognosis prediction task.

The fused tensor would be considered as the input of cox-nnet to predict the hazard ratio. We also used the partial log-likelihood function as the loss function.

### Evaluation metrics for models

We evaluated our method using two types of metrics. The commonly used log-rank p-value of Cox-PH regression was utilized as the first evaluation metric. Kaplan-Meier survival curves for two risk groups were plotted, and the log-rank p-value was calculated to assess the differences between these curves.

We also employed C-index as the second evaluation metric.The C-index is calculated based on Harrell’s C statistics and can be seen as the ratio of all pairs of individuals whose predicted survival time are ranked correctly^39,40^. A model is considered good when C-index score around 0.7, whereas a score around 0.5 means random background.

To assess the independent prognostic value of the Fusion Net score beyond standard clinical variables, multivariable Cox proportional hazards regression models were constructed. For the BRCA dataset, the model incorporated the Fusion Net score, age, and tumor stage. For the NSCLC dataset, the model included the Fusion Net score, age, gender, and tumor stage. Proportional hazards assumptions were verified using Schoenfeld residuals, and sensitivity analyses using stratified models were performed for covariates violating the assumptions.

## Electronic supplementary material

Below is the link to the electronic supplementary material.

Supplementary Material 1

## Data Availability

The datasets generated and analysed during the current study are available from the corresponding author on reasonable request.
